# Traumatic vertebral artery dissection presenting with incomplete congruous homonymous quadrantanopia

**DOI:** 10.1186/1471-2415-10-14

**Published:** 2010-05-19

**Authors:** Albert I Matti, Andrew W Lee, Celia S Chen

**Affiliations:** 1 Department of Ophthalmology, Flinders Medical Centre, Flinders Drive, Bedford Park, Adelaide, SA 5042, Australia; 2 Regional director of Stroke Medicine, Southern Adelaide Health Service, Flinders Medical Centre, Flinders Drive, Bedford Park Adelaide, SA 5042, Australia; 3 Department of Ophthalmology, Flinders Medical Centre and Flinders University, Flinders Drive, Bedford Park, Adelaide SA 5042, Australia

## Abstract

**Background:**

To describe a rare presentation of vertebral artery dissection (VAD) as a small but congruous incomplete homonymous hemianopia demonstrating use of visual field testing in the diagnosis.

**Case presentation:**

A 30 year old woman had been unwell for 4 months with difficulty focusing, vertigo, dizziness and a feeling of falling to the right. A small but congruous right inferior homonymous quadrantanopia was found on examination leading to further investigation that uncovered a vertebral artery dissection and multiple posterior circulation infarctions including a left occipital stroke matching the field defect.

**Conclusions:**

We describe an atypical case of VAD presenting with a small congruous quadrantanopia. This is a rare but significant condition that predisposes to multiple thromboembolic infarction that may be easily misdiagnosed and a high index of suspicion is required to make the diagnosis.

## Background

Vertebral arterial dissection (VAD) may occur after neck trauma [1,2], and along with carotid artery dissections may account for 25-30% of ischaemic strokes in patients <50 years [3]. Diagnosis can be difficult due to its rarity and varied symptoms. Described here-in a case of VAD resulting in multiple cerebral infarctions. The diagnosis was made after finding a small congruous homonymous quadrantanopia.

## Case presentation

A 30 year old woman became unwell after swimming with crocodiles while suspended under water in a protective cage in the Australian Northern Territory. She was vertiginous after coming out of the water and required admission to a tertiary hospital emergency department and was discharged with a diagnosis of vestibular neuronitis.

**Figure 1:**
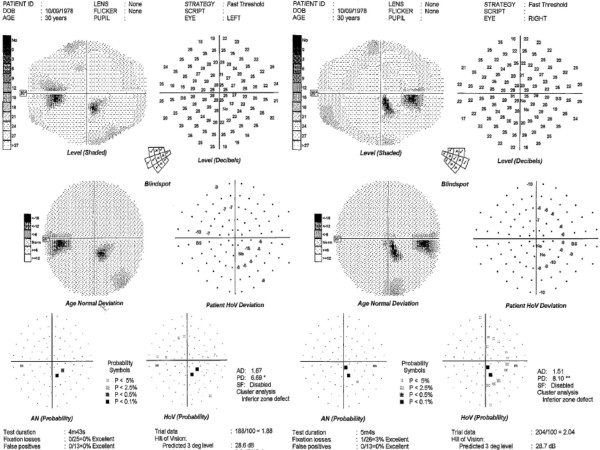
**Automated perimetry with Medmont Central 100 strategy showing a small right inferior quadrantanopia**.

**Figure 2:**
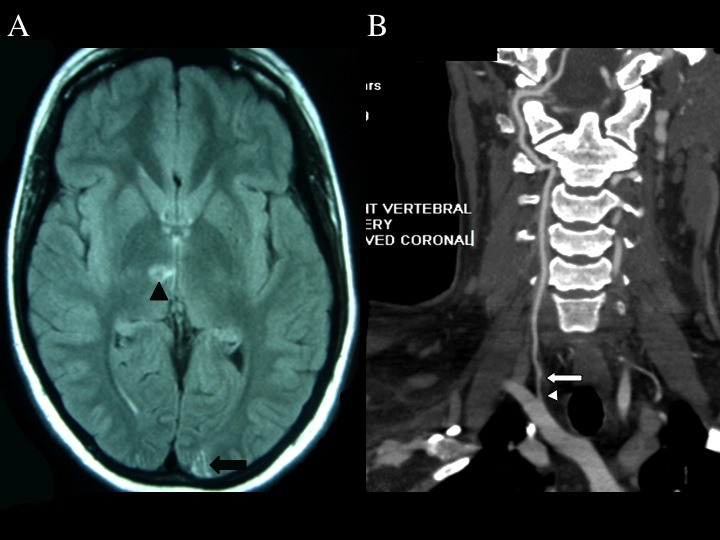
**Diagnostic imaging results**. (A) Magnetic Resonance Imaging Axial T2 FLAIR section showing a left occipital infarct (arrow) and also a right paramedian thalamic infarct (arrowhead). (B) Coronal view of the right vertebral artery on CT angiogram showing thinning of the artery (arrowhead) and shadowing of the dissected section of the vertebral artery (arrows).

She continued to experience symptoms of vertigo, difficulty focusing, dizziness and a feeling of falling to the right for over 4 months and saw various healthcare providers with no cause found. She had a plain computerized tomography of the head without contrast that was reported as normal. Eventually she was referred to a neuro-ophthalmologist for the complaint of "difficulty focusing" and automated perimetry showed a small incomplete congruous homonymous quadrantanopia (Figure 1). Magnetic resonance imaging (MRI) of the brain revealed a left occipital infarct. There were multiple areas of bilateral posterior circulation infarcts involving the cerebellum, and right thalamus (Figures 2a). Echocardiography, carotid ultrasound and hypercoagulable screens were negative. A computerized tomography (CT) angiogram showed a right vertebral dissection with both thinning and a double lumen of the vertebral artery (Figure 2b). The patient was placed on anticoagulant therapy with no further neurological symptoms.

Vertebral artery dissection (VAD) is a potentially fatal condition where disruption of the vessel wall results in thromboembolism and subsequent ischemic stroke. It may occur following neck injury resulting in neck extension, flexion or rotation [1-3]. Symptoms include neck or head pain, partial Horner's syndrome and those of ischemic stroke in its involved territory [3]. The presentation may vary from case to case. The patient likely sustained the VAD following minor neck torsion injury during swimming, followed by a shower of emboli causing multiple posterior circulation infarcts. The congruous nature of the quadrantanopia pointed to an occipital lobe infarct.

This case demonstrates the importance of ancillary imaging in making the diagnosis. CT angiography provides accurate imaging of the vessel lumen and dissection length. It can identify VAD in both large and medium vessel dissections in the neck [4]. T1-weighted MRI is an alternative but may not detect initial stages of intramural haemorrhage seen in early VAD[1]. It is for this reason that, recent studies suggest that CTA is the preferred imaging modality to identify cervical dissections, especially for VAD [5].

Management of VAD to prevent further embolic stroke is done on a case by case basis. There is no data to support the therapeutic superiority of anticoagulants over antiplatelet agents[6]. In this case the patient had no contraindications to anticoagulants including intracranial arterial dissection, large stroke, enlarging intramural haematoma and/or high risk of bleeding. The presence of multiple cerebral emboli with no contra-indication favoured the use of anticoagulants in this case.

## Conclusion

In conclusion, we describe an atypical case of VAD presenting with a small congruous quadrantanopia. This is a rare but significant condition that may be easily misdiagnosed and a high index of suspicion is required to make the diagnosis.

## Competing interests

The authors declare that they have no competing interests.

## Authors' contributions

AM carried out preparation and drafted the manuscript. CSC and AWL were the treating doctors involved in the investigation and management of the case. All authors read and approved the final manuscript.

## Consent

Written informed consent was obtained from the patient for publication of this case report.

## Pre-publication history

The pre-publication history for this paper can be accessed here:

http://www.biomedcentral.com/1471-2415/10/14/prepub
